# Biallelic *MINAR2* variant is associated with nonsyndromic severe to profound sensorineural hearing loss

**DOI:** 10.1038/s41439-025-00328-w

**Published:** 2025-10-23

**Authors:** Naif A. M. Almontashiri

**Affiliations:** 1 Center for Genetics and Inherited Diseases, Taibah University, Madinah, Saudi Arabia; 2 Faculty of Applied Medical Sciences, Taibah University, Almadinah Almunwarah, Saudi Arabia

**Keywords:** Genetic testing, Genetic testing

## Abstract

MINAR2 is essential for normal hearing by regulating cholesterol localization in stereocilia in hair cells. MINAR2 knockout results in rapidly progressive sensorineural hearing loss (SNHL) in mice and zebrafish models. Recently, biallelic variants in *MINAR2* have been reported to cause SNHL in four unrelated families with nonsyndromic severe to profound SNHL. Here we provide a second report of an additional family with SNHL. The index patient presented with nonsyndromic severe to profound SNHL. The family history was remarkable for a 20-year-old male sibling with nonsyndromic severe to profound SNHL. Both patients did not have any neurological involvement. Trio whole-exome sequencing of the index and his parents revealed a homozygous nonsense variant in *MINAR2* (NM_001257308.2:c.319A>T; p.(Lys107*) in the index. Parents were heterozygous for the same variant. This variant introduces an early stop codon and probably results in a loss of function because of the predicted nonsense-mediated decay. Our study provides the first independent confirmation of the *MINAR2*-related SNHL.

The global prevalence of sensorineural hearing loss (SNHL) is estimated to be ~6% (ref. ^1^). SNHL is the most common type of hearing loss. It can be caused by abnormalities in cochlea (in the inner ear), auditory nerve or central nervous system^2^. SNHL can be unilateral or bilateral, and the severity spectrum of hearing impairment varies from mild to profound. SNHL can result from both genetic (syndromic and nonsyndromic) and nongenetic causes. About 150 genes are associated with SNHL, with the recessive forms being the most common type associated with nonsyndromic SNHL^3,4^. Biallelic variants in the *GJB2* are the most common genetic cause of SNHL^2^. Identifying the genetic etiology can inform SNHL management and intervention^4,5^. With advances in sequencing technologies and decreasing costs, the number of genes associated with SNHL is expected to continue increasing over time.

Recently, biallelic variants in the membrane integral NOTCH2-associated receptor 2 (*MINAR2*) gene were reported to cause autosomal recessive deafness-120 (DFNB120, OMIM#620238) in 13 patients (4 unrelated families) with nonsyndromic congenital (9 patients) or prelingual (4 patients), severe to profound SNHL, consistent with mouse and zebrafish model of MINAR2 ablation^6^. Progressive SNHL was reported in four patients. In contrast to the mouse model with Parkinson’s disease-like phenotype^7^ and despite the patients’ age range of 4–80 years, none of the patients developed any neurological features. Two families were found to carry a homozygous missense variant in the Notch receptor intracellular domain (NRID), which was confirmed to disrupt the donor splice site. The other two families carried loss-of-function (LoF) variants: a nonsense variant in the NRID and a frameshift variant affecting the transmembrane domain.

MINAR2 is an endoplasmic reticulum resident protein. In humans, MINR2 is differentially expressed in brain tissues, the esophagus and reproductive organs. In mice, it is expressed in the inner ear and brain tissues^6,7^. It binds to and colocalizes with NOTCH2 in the endoplasmic reticulum^7^. This binding is required for motor function and normal hearing. In patients with Lewy body dementia, MINAR2 was shown to be downregulated in the frontal lobe brain, consistent with the phenotype of the MINAR2-knockout mice^7^. This neurological phenotype was associated with the loss of tyrosine hydroxylase-positive neurons and upregulation of α-synuclein. Interestingly, two studies reported rapidly progressive SNHL in mice and zebrafish models^6,8^. MINAR2 was shown to be essential for hearing by regulating cholesterol distribution in hair bundles, and its ablation resulted in reduced cholesterol localization in stereocilia in hair cells, indicating that MINAR2 is required for normal cholesterol homeostasis and proper distribution in hair cells^8^.

In this study, we recruited a family affected by SNHL to identify the genetic cause of the condition using whole-exome sequencing (WES). Informed consent was obtained from all the members. Ethical approval for this study was granted by the institutional review board protocol from Taibah University (approval no. TU-MLT-2019-07).

The index case is a 10-year-old male, delivered via cesarean section due to respiratory distres. He was born at full term following an uneventful pregnancy (Fig. 1A). The patient was referred to the genetics clinic from the otorhinolaryngology clinic with a history of nonsyndromic bilateral, severe to profound congenital SNHL. There is no history of seizures or visual abnormalities. His development and cognitive functions are appropriate for his age. His parents are first cousins. His 20-year-old male sibling has nonsyndromic bilateral, severe to profound congenital SNHL, and his neurodevelopmental functions are appropriate for his age. On examination, both patients appeared well with no dysmorphic features. Their growth parameters were within normal percentiles, and their systemic examination was unremarkable.

**Fig. 1:**
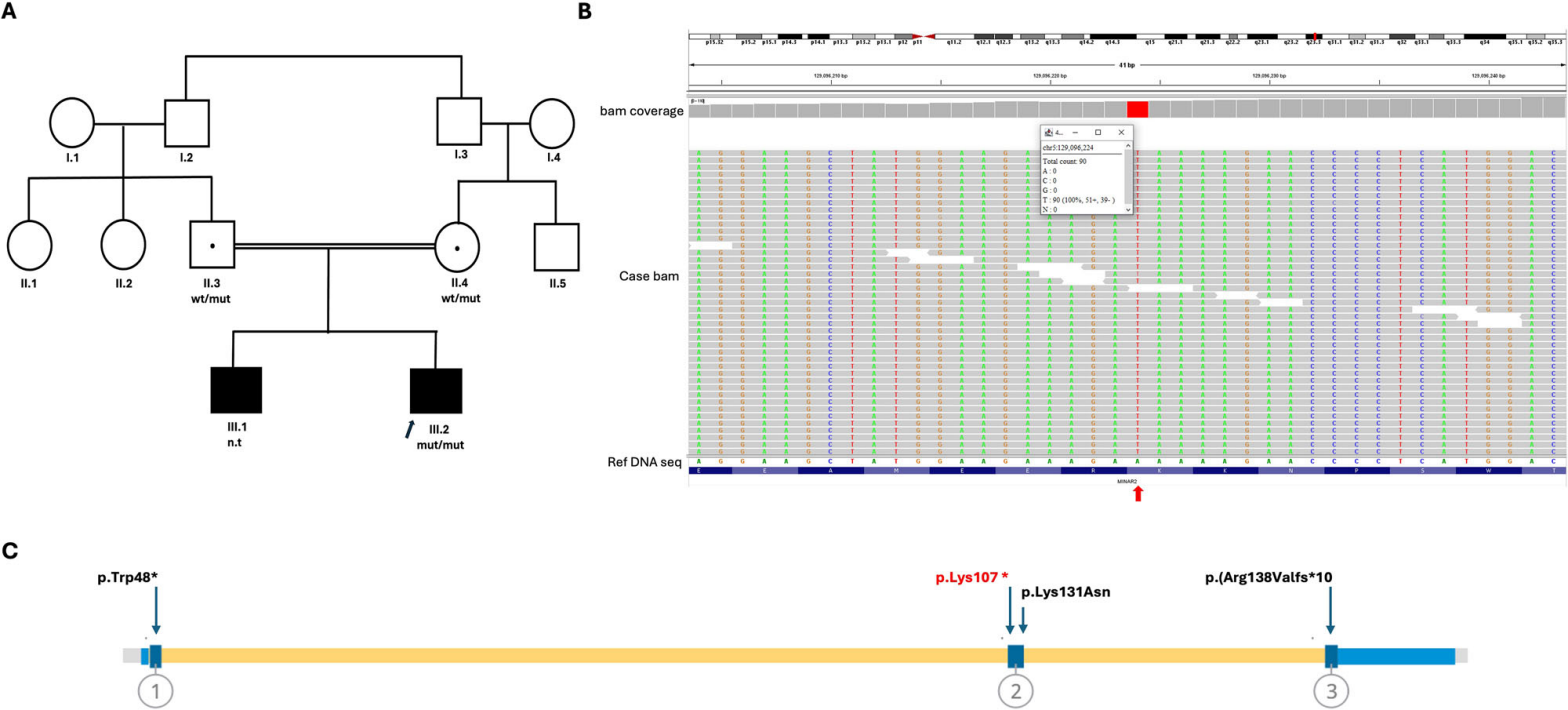
Family pedigree and exon location of the reported MINAR2 variants. **A**, **B** Family pedigree showing the segregation (**A**) and WES-based integrative genomics viewer (IGV) (**B**) of the detected homozygous nonsense variant in *MINAR2*. The red arrow shows the location of the ‘T’ variant in the bam file compared with the reference allele ‘A’ at genomic position g.129096224 (GRCh37/hg19). **C**, The *MINAR2* structure showing the exon-level location of the identified variants. The nonsense variant identified in this study is labeled in red. wt/mut, heterozygous for the variant; mut/mut, homozygous for the variant; n.t, not tested; Ref DNA seq, reference DNA sequence.

Given the family history of two affected siblings with SNHL, trio WES was performed, followed by Sanger confirmation. Testing was conducted at an external, College of American Pathologists-accredited commercial laboratory. In brief, DNA was extracted from whole blood samples drawn from the patient and his parents. The libraries were paired end sequenced on an Illumina platform with an average depth of 30×. The sequencing reads were aligned to the Genome Reference Consortium Human Build 37 (GRCh37/hg19), as well as the revised Cambridge Reference Sequence of the Human Mitochondrial DNA (National Center (NC)_012920). All variants including single-nucleotide variants and copy number variations were called using DRAGEN, Manta and in-house algorithms. Variants that were not found as homozygous in the Genome Aggregation Database (gnomAD, V4.1.0) database, or disease-causing variants reported in Human Gene Mutation Database, in the public archive of interpretations of clinically relevant variants (ClinVar) were evaluated. All potential modes of inheritance were considered. In addition, the provided clinical information and family history were used to evaluate the identified variants with respect to their pathogenicity and disease causality. Variants were classified according to the American College of Medical Genetics guidelines for variant classification in addition to Clinical Genome Resource recommendations^9^. All relevant variants related to the phenotype of the patients were considered. Mitochondrial variants with a heteroplasmy level of 15% or higher were considered.

WES revealed this homozygous nonsense variant in *MINAR2*: NM_001257308.2:c.319A>T; p.(Lys107*) in the index case, whereas the older affected sibling did not consent to genetic testing (Fig. 1A, B). The parents were carriers of the same variant. The variant was confirmed by Sanger sequencing. This variant is ultrarare, observed in a heterozygous state in 2 out of 767,859 healthy individuals in gnomAD, with a minor allele frequency of 0.0001%.

This is a nonsense variant resulting in the introduction of a premature stop codon in exon 2, upstream the previously reported missense variant (Fig. 1C). It is predicted to undergo a nonsense-mediated decay and result in a LoF of the MINAR2. This variant met the following classification criteria: very strong (null variant, PVS1) and moderate (allele frequency, PM2). According to the American College of Medical Genetics guidelines, this variant was classified as likely pathogenic.

We report a patient with bilateral, nonsyndromic severe to profound hearing loss, along with a family history of a sibling presenting with the same phenotype and disease severity. WES revealed a homozygous novel LoF variant in *MINAR2* that explains the isolated hearing loss phenotype observed in this patient. Thus, our study provides a second report and an independent confirmation of *MINAR2* association with nonsyndromic SNHL.

## HGV Database

The relevant data from this Data Report are hosted at the Human Genome Variation Database at 10.6084/m9.figshare.hgv.3550.

## References

[1] Olusanya, B. O., Davis, A. C. & Hoffman, H. J. Hearing loss: rising prevalence and impact. *Bull. World Health Org.* **97**, 646–646A (2019). 31656325 10.2471/BLT.19.224683 PMC6796666

[2] Tanna, R. J., Lin, J. W. & De Jesus, O. *Sensorineural Hearing Loss* (StatPearls Publishing, 2025). 33351419

[3] Ali, A. et al. Spectrum of genetic variants in bilateral sensorineural hearing loss. *Front. Genet.* **15**, 1314535 (2024). 38410152 10.3389/fgene.2024.1314535 PMC10894970

[4] Almontashiri, N. A. M. et al. Recurrent variants in OTOF are significant contributors to prelingual nonsydromic hearing loss in Saudi patients. *Genet. Med.*10.1038/gim.2017.143 (2018). 29048421 10.1038/gim.2017.143 PMC5929117

[5] Shearer, A. E., Hildebrand, M. S., Odell, A. M. & Smith, R. J. *Genetic Hearing Loss Overview* (GeneReviews® [Internet]. Seattle (WA): University of Washington, Seattle, 1993). 20301607

[6] Bademci, G. et al. Mutations in MINAR2 encoding membrane integral NOTCH2-associated receptor 2 cause deafness in humans and mice. *Proc. Natl Acad. Sci. USA* **119**, e2204084119 (2022). 35727972 10.1073/pnas.2204084119 PMC9245706

[7] Ho, R. X.-Y. et al. Loss of MINAR2 impairs motor function and causes Parkinson’s disease-like symptoms in mice. *Brain Commun.* **2**, fcaa047 (2020). 32954300 10.1093/braincomms/fcaa047 PMC7425422

[8] Gao, G. et al. Kiaa1024L/Minar2 is essential for hearing by regulating cholesterol distribution in hair bundles. *eLife*10.7554/eLife.80865 (2022). 10.7554/eLife.80865 PMC9714970 36317962

[9] Richards, S. et al. Standards and guidelines for the interpretation of sequence variants: a joint consensus recommendation of the American College of Medical Genetics and Genomics and the Association for Molecular Pathology. *Genet. Med.* **17**, 405–424 (2015). 25741868 10.1038/gim.2015.30 PMC4544753

